# Evaluation of adherence monitoring system using evriMED with a differentiated response compared to standard of care among drug-sensitive TB patients in three provinces in South Africa: a protocol for a cluster randomised control trial

**DOI:** 10.1186/s13063-021-05337-y

**Published:** 2021-06-09

**Authors:** Noriah Maraba, Catherine Orrell, Candice M. Chetty-Makkan, Kavindhran Velen, Rachel Mukora, Liesl Page-Shipp, Pren Naidoo, M. Thulani Mbatha, Katherine L. Fielding, Salome Charalambous

**Affiliations:** 1 The Aurum Institute, Parktown, Johannesburg, South Africa; 2 Department of Medicine and Infectious Disease and Molecular Medicine, University of Cape Town, Cape Town, South Africa; 3 Desmond Tutu HIV Foundation, Cape Town, South Africa; 4 School of Public Health, University of Witwatersrand, Johannesburg, South Africa; 5 Health Economics and Epidemiology Research Office, Johannesburg, South Africa; 6 Interactive Research and Development, Johannesburg, South Africa; 7 Stellenbosch University, Stellenbosch, South Africa; 8 London School of Hygiene & Tropical Medicine, London, UK

**Keywords:** Medication adherence, Tuberculosis, Medication monitor, Real-time medication monitoring

## Abstract

**Background:**

South Africa has achieved drug-susceptible TB (DS-TB) treatment success of only 77% among people with new and previously treated TB. Alternative approaches are required to improve medication adherence and treatment completion to limit transmission, TB relapse and the development of resistance. This study aims to implement and evaluate the use of adherence medication monitors (Wisepill evriMED 1000) with a differentiated response to patient care, among DS-TB patients in three provinces of South Africa.

**Methods:**

In total, 18 public health clinics across three provinces were selected. Clinics were randomised to intervention or standard of care clinics. In each clinic, approximately 145 DS-TB patients are being enrolled to reach a total of 2610. All patients have their daily adherence monitored using medication monitors. In the intervention arm, patients are receiving medication monitor reminders and differentiated care in response to adherence data. This weekly review of daily real-time monitoring will be undertaken from a central database. The differentiated care model includes automated SMS reminders with a missed dose, research staff-initiated phone call to the patient with a second or third missed dose, a home visit if four or more doses are missed, and motivational counselling if four or more doses are missed repeatedly. Fidelity of the intervention will be measured through process evaluation. Patients in control clinics will receive medication monitors for adherence tracking, standard of care TB education, and normal clinic follow-up procedures. The primary outcome is the proportion of patients by arm with >80% adherence, as measured by the medication monitor. The feasibility and acceptability of the intervention will be assessed by in-depth interviews with patients, stakeholders, and study staff. A cost effectiveness analysis of the intervention and standard of care clinics will be conducted.

**Significance:**

This trial will provide evidence for the use of an intervention, including medication monitors and differentiated care package, to improve adherence to TB treatment. Improved adherence should also improve TB treatment completion rates, thus reducing loss to follow-up rates, and TB relapse among people with TB. The intervention is intended to ultimately improve overall TB control and reduce TB transmission in South Africa.

**Trial registration:**

Pan African Trial Registry PACTR201902681157721. Registered on 11 February 2019.

**Supplementary Information:**

The online version contains supplementary material available at 10.1186/s13063-021-05337-y.

## Introduction

Globally, an estimated 10 million people with TB were identified with new and previously treated tuberculosis (TB) in 2018 [1]. Of the 6.4 million people with TB, who started on treatment in 2017, treatment success was reported in 85% [1]. South Africa reported treatment success rates of 77% among all people with new and previously treated TB as well as 75% among HIV-positive people with TB [1]. Poor medication adherence contributes to lower treatment completion among people with TB [2]. Poor medication adherence also contributes to an increased rate of TB relapse [3].

Systematic reviews show that the traditional approach to facility-based directly observed treatment (DOT) does not improve treatment completion, microbiological cure, disease relapse rates, and rates of acquired drug resistance when compared to self-administered therapy [4–6]. Facility-based DOT is time-consuming, is highly resource intensive, and creates barriers to retention in care and treatment completion. It is also inconvenient to people living with TB and results in loss of income and employment [7, 8]. Alternative and innovative approaches are required to improve medication adherence and treatment completion to limit transmission, TB relapse, and the development of drug-resistance.

A number of digital health adherence technologies have been explored recently including the electronic medication monitor (as in this study), the 99-DOTs (a cardboard sleeve which requires patients to send a short message services [SMS] or make a missed call after taking a dose) [9], and video-supported treatment [10] (smart phone based technology using video to record medication intake). A cluster randomised trial of 4500 TB patients in rural and urban China showed improved medication adherence in patients randomised to the medication monitor compared to SMS messaging or the standard of care [11]. The medication monitor was well accepted by patients and providers [12]. A differentiated care approach, one of the pillars of the End-TB strategy, defined as a client-centred approach that simplifies and adapts services along the care cascade, may better serve the needs of patients and reduce the burden on the health system and has the potential to improve medication adherence [13]. A further study to evaluate the use of medication monitor, this time with real-time reporting and with differentiated care, is being conducted in China [14]. Electronic medication monitors have not been formally evaluated in drug-susceptible TB (DS-TB) patients in South Africa. The use of these electronic monitors could assist the country in reaching the global TB plan target of 90% successfully completing their treatment. Monitoring of the adherence data will assist in identifying who needs more support under a differentiated model thereby directing scarce resources to them. In this trial, we will evaluate whether implementation of a medication monitor (smart pill box; Wisepill evriMED 1000 device), coupled with daily monitoring and differentiated care, is able to improve adherence and reduce poor outcomes in patients with DS-TB treatment.

## Methods

The study protocol has been reported in accordance with the Standard Protocol Items: Recommendation for Clinical Interventional Trials (SPIRIT) guidelines (additional file 1 for details).

### Study design and setting

The trial is parallel group, cluster-randomised taking place in six clinics in each of three provinces in South Africa: Gauteng (Ekurhuleni district), Western Cape (Klipfontein and Mitchell’s Plain districts), and Kwa-Zulu Natal (eThekwini district). The study is evaluating if the intervention is superior to the control treatment. Clusters were defined as public health clinics with at least 200 TB registrations in 2017. Adult HIV prevalence in the general population is 18.2% in Kwa-Zulu Natal, 12.5% in Gauteng, and 8.9% in Western Cape province [15]. Prior to randomisation, we considered urban or rural location of clinic, total number of patients starting TB treatment per month, and treatment outcomes in the last 12 months. Clusters were randomised 1:1 to intervention or standard of care arm using restriction to ensure that each province has at least one intervention and one standard of care clinic [16]. Randomisation was conducted by a computer programme (Stata) which generated all possible randomisations (48,620). Restrictions were applied to eliminate randomisations for which facilities in the two groups were not balanced by province (0–1 or 5–6 facilities from each province per arm) which left 35,000 possible options. Ten thousand of the randomisations were randomly selected for convenience and listed in an Excel spreadsheet, labelled as 0000–9999. Clinic/DOH representatives present at the randomisation meeting chose 4 golf balls (each numbered 0,1,… 9) to select a randomisation option. Unfortunately, following the randomisation though before participant enrolment had started, two clinics, from each arm (same province), had to withdraw due to ongoing TB studies. These were replaced by two clinics in the same province and were allocated to the intervention and control arm at random.

### Study population and eligibility criteria

The study is recruiting all adults aged ≥18 years and children aged 2–17 years with clinically or microbiologically diagnosed DS-TB. The inclusion criteria include initiating TB treatment within the last 14 days, adults and caregivers of children who are willing and able to provide informed consent, and children aged 7–17 years old who are willing and able to provide informed assent. Patients need to be willing to use the medication monitor; agree to be followed-up with text messaging, phone calls, and home visits; live within the study catchment area; and be willing to inform the study team of any change of address during the treatment as well as follow-up period. We expect to enrol approximately 2610 DS-TB patients and follow them up for 18 months post enrolment. The study started enrolment in May 2019 and will complete follow-up in December 2021. This manuscript could not be submitted for consideration before enrolment commenced as the methodology had to piloted and refined during the early phases of the enrolment period.

### Description of study arms and procedures

#### Intervention clinics

Enrolled patients receive the differentiated care adherence package. This includes standard patient education and provision of medication monitor programmed to alert patients (using sound and a light on the box) on a daily basis as a reminder to take their medication (Table 1). Patients are educated on how to use the medication monitor by the study team. The medication monitor transmits a daily signal to the Wisepill system (system), referred to as heartbeat, to indicate that it is working properly. There are also additional reminders for monthly dispensing visits and the device uploads data in real-time to a central database that allows for weekly review by research staff. Weekly automated reports are generated from the system that lists patients who had missed any doses and those whose devices are not sending a signal correctly. A step-wise approach to missed doses is then implemented that includes phone calls, home visits, and motivational counselling for those who consistently miss medication doses (Table 2). Automated SMS reminders are sent to any patient in the intervention with a missed dose. A second or third missed dose in a week triggers a research staff-initiated phone call to the patient, and four or more missed doses in a week trigger a home visit. Phone calls and home visits are scripted. Should four or more doses be repeatedly missed, additional adherence measures such as motivational counselling is done. When participants visit the clinic for their usual dispensing visit, the research staff shows the participant their own data and discusses their medication adherence history.

**Table 1:** Description of scheduled study visits

***Study Visit*** ***Month (M) and*** ***Day (D)***	***D0*** ***(Enrolment)***	M1	M2	M3	M4	M5	M6	M9	M12	M15	M18
Informed consent	**√**										
Eligibility criteria verified	**√**										
Of those eligible:											
Locator information	**√**	**√**	**√**	**√**	**√**	**√**	**√**	**√**	**√**	**√**	**√**
Brief medical history	**√**										
Risk assessment for TB	**√**										
Economics questions	**√**										
Stigma scale	**√**						**√**				**√**
Facility dispensed treatment	**√**	**√**	**√**	**√**	**√**	**√**					
Education on the medication monitor and monitor provided to patient	**√**										
Medication monitor configured to have visual and audio reminders—intervention arm only	**√**										
Participants’ experience using medication monitor and adherence discussion for intervention—intervention arm only		**√**	**√**	**√**	**√**	**√**	**√**				
Social harm							**√**		(√*)		
Pre-treatment, two and five months routine specimens abstracted from record							**√**				
Treatment completion history abstracted from routine records							**√**				
TB Symptom screen**								**√**	**√**	**√**	**√**
Research clinic visit								**√**	**√**	**√**	**√**
Sputum culture and GXP	**√**^(^*^)^						**√**	**(√)**	**(√)**	**(√)**	**√**

*GXP* Gene Xpert MTB/Rif

(*) collect culture for those who were diagnosed via chest radiograph or clinically or have negative GXP result; **TB investigations performed if participant is symptomatic for TB; (√) sputum collected for culture and GXP if TB symptoms are reported at that visit; (√*) only done if social harm case report form was not completed at month 6

**Table 2:** Description of the differentiated care in the intervention arm

Weekly adherence	Weekly doses missed	Intervention
85%	1	Same day SMS reminder (automated)
45–84 %	2 or 3	Voice call by research staff
<45%	4 or more	Home visit and/or clinic visit
	Greater than 1 week with 4 or more missed doses	Recall for other adherence measures, e.g. motivational counselling.

#### Standard of care clinics

Enrolled patients are provided with a return date for collection of repeat medication in 30 days. Patients are also provided with the medication monitor and educated on its use by the study team (Table 1). The medication monitor records data on box opening and is not configured to have visual or audio reminders for intakes or refills. The medication monitor transmits a daily “heartbeat” signal to the system to indicate that it is working properly. In contrast to the intervention clinic patients, data collected on the system is not reviewed during follow-up study visits. Patients receive counselling regarding their TB treatment as per standard of care only. Neither the research staff nor the patient accesses the medication monitor information at follow-up study visits.

#### Follow-up

Follow-up of all patients in intervention and standard of care arms continues every 3 months until 12 months after completion of treatment (approximately 18 months after enrolment) (Table 1). Patients who die, declared as loss to follow-up during treatment by facility staff, or move out of our study area will be withdrawn from study. At each follow-up visit, TB symptom screening will be performed and, if required additional investigations such as sputum tests, will also be conducted. Patients are reimbursed ZAR 50 for travel costs for each visit. All patients are requested to provide sputum specimens for culture testing at 6 months from enrolment and 12 months post TB treatment completion (18 months from enrolment).

### Qualitative evaluation

The feasibility and acceptability of the intervention will be assessed by conducting at least 60 in-depth interviews with patients who received the intervention, and at least six stakeholders and study staff who implemented the intervention. Patient interviews will be done post enrolment across varied follow-up times to allow for variation regarding the use of medication monitors. They will also be selected according to their gender, age and how adherent they are according to the weekly analysed adherence data (adherence category). These patients will be reimbursed R150 for their time. For feasibility of the intervention, we will explore the following: how easy it was to use the medication monitors, concerns about inadvertent disclosure of TB status with having a medication monitor for medication, and how useful patients found the SMS reminders, calls and home visits. Key stakeholders at provincial and district level will be interviewed to understand the motivators and challenges to integrating this model of care into the current health system and how the use of this technology could influence policy change. For acceptability, we will explore appropriateness of the intervention, issues encountered, and perceptions of benefit from both patients and providers.

### Economics evaluation

The cost-effectiveness analysis of the intervention will entail estimating all direct and indirect costs from the intervention and standard of care clinics. Costing will be based on a societal perspective. All costs related to the resources utilised in delivering the intervention such as personnel, equipment, supplies, and training will be gathered using a bottom-up costing approach. Personnel costs associated with time spent conducting their daily activities will be acquired using a survey and time and motion methods and timesheets. Time spent on voice calls to patients will be estimated using itemised billing records from the service provider. Patient costs will be estimated using a questionnaire at enrolment, 2 and 6 month’s visits, and compared in both trial arms to identify patient cost drivers. We will calculate the incremental cost-effectiveness ratio for the clusters per treatment arm.

### Ethics approval

The study has ethical approval from the University of Witwatersrand (Ref 180705) and the University of Cape Town (Ref 452/2018), as well as, from the London School of Hygiene & Tropical Medicine (Ref 16107). It also has approval from the health management of the three Provincial Offices, as well as City of Cape Town, eThekwini and Ekurhuleni district research committees. The study has a Trial Steering Committee that meets biannually to discuss study progress. The trial steering committee is comprised of six members and these are the following: PI of the study, Western Cape regional PI, National Department of Health representative, South African-based TB expert, and two internationally based TB experts. The steering committee is responsible for providing expert oversight of the trial, monitoring recruitment as well as follow-up rates and reviewing strategies from the investigators to deal with problems, providing recommendations as to the future continuation of the trial, reviewing regular data reports of the trial progress, and lastly approving external or early internal requests for release of data or subsets of data or samples including clinical data and stored biological samples. Written, informed consent is being sought from all potential participants by study staff (research assistant) before taking part in the study. Assent is also being obtained from participants aged 7–17 years.

### Trial outcomes

The primary study endpoint is medication adherence which will be measured as the proportion of patients by arm with >80% adherence, as measured by the medication monitor. For those lost to follow-up during treatment, we will assume no drug intake (100% non-adherence) for the period from the date of being lost to follow-up to the date of scheduled treatment completion. Secondary endpoints will be successful outcome at the end of treatment and unfavourable outcomes at 18 months post enrolment. Treatment success will be measured as the proportion of patients by arm that either have a documented cure or treatment completion by 210 days following commencement of TB treatment. A TB culture will be performed at the end of treatment to confirm response to treatment. TB recurrence will be determined through incident TB cases in the year following completion of DS-TB treatment. Unfavourable outcome at 18 months after enrolment will include on-treatment lost to follow up, death, treatment failure, and treatment recurrence. Fidelity of the intervention will be measured by reporting on percentage of patients who were supposed to receive an SMS or phone call who did and percentage of patients who received counselling when required. We will also report on percentage of boxes with failures. We will also report on percentage of patients withdrawing from study due to refusal of using digital adherence technology.

Qualitative outcomes will be on appropriateness and perceptions of patients as well as providers involved with the implementation of the intervention. We will also report on reaction to use of the medication monitor as well as the suitability of this medication monitor to participant living conditions, the motivators of using the medication monitor to take treatment, and challenges to integrating the differentiated model of care into the current health system. We will further explore differences across patients by age, category of adherence, and location.

Economic outcomes will be based on cost per person with >80% adherence to DS-TB treatment, cost per person with TB who successfully completed DS-TB treatment, proportion experiencing catastrophic costs, and cost drivers and incremental cost-effectiveness ratio.

### Study outcomes’ definitions

Treatment completed is defined as patient who has completed their treatment without evidence of failure but without any record of a negative smear or culture result in the last month of treatment or any time in previous occasion.

Cured is defined as pulmonary TB patient with a positive smear, Xpert MTB/Rif or culture result (bacteriologically confirmed TB) at the beginning of treatment who has a negative smear microscopy or culture result at the last month of treatment.

Lost to follow-up is defined as a patient whose treatment has been interrupted for more than 2 months.

Treatment failed is defined as a patient whose sputum smear or culture is positive at month 5 or later during treatment.

TB recurrence is defined as a patient with a positive culture result at any time during the 12 months follow-up period post treatment completion.

Unfavourable outcome at 18 months post enrolment is defined as treatment failure, lost to follow-up and death during treatment, and recurrence after end of treatment.

### Sample size considerations

Sample size calculations were conducted accounting for the clustered design [16]. Throughout we have assumed a harmonic mean of 145 TB patients/cluster, nine clusters per arm and a two-sided type I error of 5%.

Proportion with >80% adherence: Assuming a percentage with non-adherence in the standard of care arm of 30% and coefficient of variation of variation of 0.12, we will have 90% power to detect a 40% reduction in the endpoint in the intervention arm. For a successful outcome at the end of treatment of 80% in the standard of care arm and a coefficient of variation of 0.06, we will have 90% power to detect an increase to 90%. For unfavourable outcome 18 months after enrolment (defined as on-treatment lost to follow-up, death, treatment failure, and recurrence) of between 13 and 20% in the standard of care arm and a coefficient of variation of 0.25, we will have at least 80% power to detect a 40% reduction in the intervention arm.

### Data management and analyses

Research staff were trained to capture all data into web-based database which is encrypted to ensure compliance to the South Africa’s Protection of Personal Information Act (POPI). If Internet connection is not available, a paper-based system is implemented and data captured into the web-based system once Internet connectivity is restored. Documents containing personal identifiers will be kept in lockable cabinets that are only accessible by study staff.

The database access is restricted with an encrypted password. All fields have automated system checks, which verify missing, range, and future dates. Any personal identifiers will be removed from the analytic dataset prior to the data analysis phase of the study. The data management team carries out a centralised statistical monitoring. Baseline characteristics data will summarised at individual level for all participants in the two arms. The individual data will include age, gender, ethnicity, education, marital status, number of people they live with, special risk factors for TB, TB history, HIV status, antiretroviral status, CD4 count, and viral load. We will also report on TB treatment category, sputum results, smoking, alcohol and recreational drugs use, and TB related stigma at three time points which are at the start of taking TB treatment, completion of TB treatment, and end of 18 months follow-up. The study is also collecting data on social harm during the course of treatment at the end of TB treatment and adverse events will be reported to the Ethics committees. To answer our main objective, we will measure adherence using data from the medication monitors. A box opening any time during the day will be taken as a proxy for medication intake. Adherence will be calculated by dividing the number of days when pills were taken over number of days that pills were supposed to be taken to generate an adherence proportion for each individual enrolled. We will then generate a binary variable for each participant based on ≥ 80% adherence.

Analysis will be conducted at cluster-level due to the small number of clusters [16]. For each cluster the proportion of participants with ≥80% adherence is measured and the log(proportion) compared by study arm using a t test. We will also conduct an adjusted analysis for the intervention effect, adjusting for imbalances of individual-level variables at baseline, using a two-stage approach [16]. We will fit a logistic regression model at the individual-level including any baseline variable which look imbalanced by study arm and variables which we anticipate to be associated with poor adherence. The expected outcome for each individual is calculated and summed at the cluster-level. The log of cluster-level residual (expected number of outcomes with the observed number of outcomes) is compared by study arm using a t test. We plan to also specify a limited number of subgroups and endpoint(s) they relate to. Subgroups will be measured at the cluster-level (e.g. province) or individual-level (e.g. sex, education, stigma-level). We will then estimate the intervention effect in each subgroup. A full statistical analysis plan will be developed before study completion.

For the secondary objectives, we will generate a binary variable indicating whether enrolled participants had successfully completed six months of TB treatment or not. Unfavourable outcomes will be measured by generating a binary variable indicating whether enrolled participants had an unfavourable outcome 18 months after enrolment.

A detailed statistical analysis plan will be written prior to any analyses. The trial investigators will have access to the final dataset.

For the qualitative component, audio-recordings and process notes will be transcribed and where necessary translated prior to analysis. Data will be analysed using QSR NVIVO 10 qualitative analysis software and manual reviewing. Thematic analysis using deductive and inductive approaches will be used to describe the themes. A codebook will be created using the deductive themes and updated with any new themes that develop from the transcripts. Saturation of themes will be assessed during study implementation by frequent review of the transcripts. For reliability, the original transcript and codebook will be sent to two independent reviewers.

For economics evaluation, all time and motion data will be collected onto a paper form and captured onto an Excel workbook by trained research assistants. The health economist will review the data on a regular basis and clarifications requests made promptly to avoid recall bias. Patient costing data will be collected on case report forms and entered into a password-protected database created in REDCap database. Data will be exported from REDCap into STATA (version 14, StataCorp LP, College Station, Texas) for analysis.

## Discussion

The study aims to evaluate whether implementation of medication monitors with daily remote monitoring and differentiated care is able to increase the proportion of patients with ≥ 80% adherence to drug sensitive TB treatment. Although medication monitors have been used to measure adherence in HIV treatment patients, this study is among the few studies investigating adherence using medication monitors among drug-sensitive TB patients in sub-Saharan Africa [17]. The study is also one of the first to evaluate differentiated care following information from an adherence technology. The medication monitor offers a real time documentation of medication intakes, which allows ability to monitor medication adherence whenever required. This approach will also allow for a further discussion of why non-adherence occurred when participants return to facility for refills. The differentiated care approach enables staff to follow-up on a weekly basis on participants that have missed doses allowing for timely action on missed doses unlike waiting for patients to return to facilities at end of the month as it is currently done in routine practice. The approach will also assist us in identifying people with TB who may need more support when on TB treatment.

Other digital adherence technologies that have been explored for TB medication adherence include 99-DOTS, which requires patients to give a missed call to a toll free number when the pills are removed from the medication blister pack when taking a dose. The disadvantage of using 99-DOTS is that treatment support before treatment intake is not provided but only done once a patient misses a dose [9]. Another technology that has been explored is video-supported treatment where patients take a video of themselves ingesting TB treatment and sending it to health care providers to view later [18]. The disadvantage with using this technology is that it requires that a patient have a smartphone and have access to Internet connection. Other criticisms have been that it is an intrusive and patronising method. Both these technologies require some effort from the patient to either give a missed call, send an SMS to the toll free number for 99-DOTS, or take a video of themselves ingesting medication. Furthermore, 99-DOTS sleeves have to be customised to the different medication blister packs.

The advantages of the medication monitor over the other technologies is that it has both visual and audio medication reminders and monthly refill reminders. The monitor software also automatically sends text messages to patients if a patient does not take their medication at the scheduled time. All these features attempt to support patients when taking treatment [19]. With differentiated care based on adherence monitoring, one may be able to counter issues, related to fear of side effects, lack of disclosure, lack of support, etc., through additional motivational counselling.

There are some operational challenges that need to be overcome to implement the study. To implement the intervention, staff need access to a computer/portable device to be able to assign a device to a patient. Having a computer in the TB consultation rooms is not the current practice in South Africa and we will therefore provide electronic tablets loaded with data to our study team so this can be done. The research team is operationalising the intervention and they require cell phones and airtime to be able to call patients who missed their doses, these will also be provided by the study. The configuring of each device also requires access to an Internet connection. Most facilities in South African do not have free Internet access available and the study must therefore provide data to the study team. Reports that will be used to implement the differentiated care will not be automatically generated, so a dedicated person needs to download and generate them weekly. One of the study staff per province will be responsible for ensuring that this is done. During the follow-up period that patients are still taking their TB treatment, we run a risk of some patients losing their medication monitors. If this occurs, the study will replace the medication monitors.

### Limitations

One limitation of the study is that intake notification through opening of the box does not mean that a patient has ingested their medication. We might get instances where the system has recorded an opening as an intake but the patient has not ingested the medication and vice versa. We will not perform plasma or urine drug concentration tests to check objectively on dosing. However, there is evidence in HIV patient populations in South Africa that electronic adherence monitoring monitors are a good measure for adherence [20].

A second limitation is that most TB patients in South Africa have HIV co-infection but the current study is not monitoring medication adherence for both diseases. While the differentiated care strategy is labour intensive, the study will allow us to evaluate which elements are more feasible and practical and which are more effective. The HIV community in the country is already using a differentiated care model to address non-adherence in patients [21]; therefore, use of this approach in TB care might bring opportunities for integrated patient management. We are assuming high levels of acceptability of the medication monitor; this will be assessed in the qualitative aspects of the study. This might also introduce some population selection bias in our study as only people that are willing to use the medication monitor will be enrolled and not everyone with TB who are started on TB treatment in the facilities that we are recruiting from.

### Strengths

The strength of this study is that it is a pragmatic trial, taking place in 18 clinics across three provinces of South Africa reflecting rural and urban settings, with varying TB and HIV burdens. In addition, the study is being conducted within the routine programme and care is likely to be influenced by the routine setting, although research staff may support some activities. We are using trained lay workers to educate participants about their treatment, which is a strength, and if found to be successful, the intervention could be implemented without requiring specialist highly trained staff. The inclusion of the socio-behavioural and economic evaluations will allow implementation issues to be explored allowing for easier implementation if the intervention is found to be successful.

### Dissemination

Our study findings will be shared with the facility managers and district officials where the study took place, as well as provincial and national Department of Health stakeholders. The study is part of the national DISRUPT TB Innovations Consortium in South Africa through which results will be shared with other TB researchers, non-governmental organisations, and provincial and national stakeholders. In addition, findings will be disseminated in both local and international conferences, and published in peer-reviewed journals. Authorship on publications generated for this trial will be granted to individuals who have contributed substantively to the design, conduct, and interpretation of trial findings as well as reporting of the trial. A de-identified participant-level dataset will also be made available after journal publication.

## Conclusions

The current study will report on the feasibility, acceptability, and cost-effectiveness of using remote medication monitors with a differentiated care approach to support DS-TB patients within the South African context. If this intervention is successful, a larger implementation project will be needed to further understand the implementation challenges of rolling out the intervention at a wider scale.

## Trial status

The protocol is version 5.0, dated 25 February 2020. The trial started recruitment on 17 May 2019. The recruitment to trial is ongoing until December 2020 and follow-up is expected to be completed on 30 December 2021.

## Supplementary Information

**Additional file 1.** SPIRIT checklist.

## Data Availability

Not applicable

## Abbreviations

Medication monitor: Wisepill evriMED 1000 device
TB: Tuberculosis
DS-TB: Drug-susceptible TB
